# Analysis of Item‐Level Disagreement Between Researchers and ChatGPT on the PEDro Scale for Assessing the Methodological Quality of Experimental Studies

**DOI:** 10.1111/jebm.70145

**Published:** 2026-06-30

**Authors:** Juan Antonio Valera‐Calero, Germán Monclús‐Díez, Isidro Fernández‐López, Mónica López‐Redondo, Sandra Sánchez‐Jorge, Jorge Buffet‐García, Kadriye Yasemin Usta‐Ayanoğlu, Umut Varol

**Affiliations:** ^1^ Department of Physiotherapy Universidad Complutense de Madrid Madrid Spain; ^2^ Faculty of Health Sciences Universidad Francisco de Vitoria Pozuelo de Alarcón Spain; ^3^ Tropico Software Istanbul Turkey; ^4^ Department of Computer Science Technische Universität Darmstadt Darmstadt Germany; ^5^ Grupo De Investigación De Alto Rendimiento En Evaluación Multidimensional y Tratamiento del Dolor Crónico Universidad Rey Juan Carlos Alcorcón Spain

1

Physiotherapy research has grown substantially over the last three decades, not only in raw publication volume but also in its relative contribution to biomedical and rehabilitation science [1]. A PubMed/MeSH‐based analysis of trends from 1995 to 2015 documented that physiotherapy papers increased from 0.54% to 2.37% of all human health research and from 38.2% to 58.7% of physical rehabilitation research [2]. This expansion has coincided with a marked evolution in the types of studies being published. Systematic reviews (SRs), essentially nonexistent in 1995, rose almost linearly to comprise 14.6% of physiotherapy publications by 2015, while randomized controlled trials (RCTs) also grew from 45.1% to 59.4%. By 2015, these two core study designs made up approximately three‐quarters of all publications in this field, reflecting an increased emphasis on producing and synthesizing higher‐level evidence [2]. Against a backdrop of rapidly accelerating biomedical literature, these patterns suggest an ongoing need for efficient, reliable approaches to evidence synthesis and to the critical evaluation of the internal validity of primary trials.

Methodological rigor in SRs depends on transparent, reproducible procedures and explicit assessment of risk of bias in included studies. The PRISMA 2020 update provides reporting standards for these steps, reinforcing the need for clearly defined eligibility criteria, robust identification and selection methods, and transparent appraisal of study quality [3, 4]. In this regard, the PEDro scale is one of the most popular tools to assess the methodological quality of experimental studies [5]. The scale covers 11 items, with 10 contributing to a 0–10 score, assessing core domains such as randomization, allocation concealment, baseline comparability, blinding of relevant parties, adequate follow‐up, intention‐to‐treat analysis, between‐group comparisons, and reporting of point estimates with measures of variability; eligibility criteria are recorded but not included in the total score [6, 7].

Recent work has highlighted the growing role of artificial intelligence (AI) in SR processes. For instance, van Dijk et al. described the use of ASReview to prioritize records [8]. Nevertheless, the potential value of AI for assessing methodological quality of primary physiotherapy trials has not been clearly established. In this context, the aim of this study is to analyze the disagreement between an AI system and the researchers who reported scores for all individual items of the PEDro scale in their SRs.

An observational cross‐sectional study was conducted. Rather than identifying experimental studies and scoring them using the scale, we used PEDro item scores as reported in published SRs since they have already undergone multiple quality‐control steps (i.e., independent dual appraisal by review authors, reconciliation by consensus, third‐party adjudication when needed by methods experts or librarians, external peer review, and final editorial oversight). Therefore, leveraging these peer‐validated ratings was expected to improve the transparency and reproducibility of our reference standard compared with generating new investigator scores. To further strengthen methodological credibility, we restricted data extraction to SRs published in journals indexed in the Journal Citation Reports, given concerns that non‐indexed or predatory journals may not ensure robust peer review or quality appraisal processes [9].

Eligible SRs were identified through a predefined PubMed search combining MeSH terms and free‐text terms: (“Physical Therapy Modalities”[MeSH] OR “Physiotherapy” OR “Physical therapy”) AND (“PEDro scale” AND “methodological quality” AND “Systematic Review”[MeSH]). Results were limited to the 10 years preceding the search (2015‐2025) and to SRs with or without meta‐analysis. Narrative, umbrella, scoping, rapid reviews, and other non‐systematic review types were excluded, as were reviews authored by members of the current research team. After full‐text screening, studies were included if they were written in English, published in JCR‐indexed journals, reported item‐level PEDro scores, and explicitly stated that at least two authors independently applied the scale and resolved conflicts via consensus and third‐party arbitration if needed.

Sample size was calculated according to Walter et al.’s method [10], informed by prior PEDro reliability evidence [5]. Assuming a minimum acceptable reliability of 0.70 [11], an expected ICC of 0.80, 80% power, and a 5% significance level, at least 148 trials with PEDro scores were required. From 690 initial search records, SRs were randomly selected and sequentially included until the minimum number of eligible trials was achieved.

Seven researchers contributed to data collection and oversight. One investigator performed the search and identified eligible reviews, another downloaded and coded included trials, and another extracted the 11 item scores and total PEDro scores from the reviews for the reference dataset. Independently, blinded assessors prompted ChatGPT (GPT‐5, OpenAI, 2025) once per article to rate them using standardized instructions and a consistent prompt requesting item‐level scoring with brief justifications (Supplementary Material). The requirement for justifications enabled adjudication of the likely source of disagreement when item‐level discrepancies arose. Statistical analyses were conducted by an investigator blinded to score sources, under supervision of senior team members [12, 13]. Finally, the list of the PEDro items on which the AI and the SRs disagreed was examined by the research team, and adjudicated which source (AI or SR) was correct by consensus.

Ten SRs fulfilled the inclusion criteria (Supplementary Material), contributing 243 randomized controlled trials assessed with the PEDro scale, with each review covering ranging from 6 to 120 trials. The included reviews were diverse in both publication venue and clinical focus, published in diverse journals from different publishers across sports and musculoskeletal physiotherapy, neurological rehabilitation, and oncology/lymphatic‐related rehabilitation.

For the analytic dataset, 16 trials were removed due to documentation and traceability issues that prevented confident verification or linkage between trials and reported PEDro scores. Exclusions were attributable to inaccessible full texts (*n* = 4), trials listed as included in the review but not found in the reference list (*n* = 2), citation mismatches between PEDro records and review bibliographies that hindered unambiguous identification (*n* = 7), and conference proceedings that did not provide sufficient methodological detail for confirmation.

Results revealed that overall, the AI tended to rate trials more favorably than researchers (Table 1). At the item level, agreement was generally high across most domains (69.2% to 94.7%), with the lowest concordance observed for Item 1. However, Item 1 has no impact in the total score as total scores are calculated as the sum of items 2 to 11. In contrast, agreement was strongest for Items 2, 6, 10, and 11 (>93% agreement), suggesting that AI and human reviewers converge more consistently on these methodological features despite the AI's tendency to score more positively overall.

**TABLE 1:** Item‐level analyses for researchers and artificial intelligence scores to determine the methodological quality using the PEDro scale.

Variables	Item 1	Item 2	Item 3	Item 4	Item 5	Item 6	Item 7	Item 8	Item 9	Item 10	Item 11	Total (0–11)
Systematic review scores (*n* = 227)												5.66 ± 1.76
No, *n* (%)	73 (32.2)	9 (4.0)	147 (64.8)	39 (17.2)	184 (81.1)	207 (91.2)	136 (59.9)	86 (37.9)	155 (68.3)	6 (2.6)	16 (7.0)	
Yes, *n* (%)	154 (67.8)	218 (96.0)	80 (35.2)	188 (82.8)	43 (18.9)	20 (8.8)	91 (40.1)	141 (62.1)	72 (31.7)	221 (97.4)	211 (93.0)	
Artificial Intelligence scores (*n* = 227)												6.54 ± 1.61
No, *n* (%)	9 (4.0)	15 (6.6)	161 (70.9)	42 (18.5)	172 (75.8)	213 (93.8)	141 (62.1)	71 (31.3)	171 (75.3)	12 (5.3)	5 (2.2)	
Yes, *n* (%)	218 (96.0)	212 (93.4)	66 (29.1)	185 (81.5)	55 (24.2)	14 (6.2)	86 (37.9)	156 (68.7)	56 (24.7)	215 (94.7)	222 (97.8)	
Agreements, *n* (%)	157 (69.2)	213 (93.8)	203 (89.4)	194 (85.5)	205 (90.3)	215 (94.7)	196 (86.3)	188 (82.8)	199 (87.7)	215 (94.7)	212 (93.4)	
Disagreements, *n* (%)	70 (30.8)	14 (6.2)	24 (10.6)	33 (14.5)	22 (9.7)	12 (5.3)	31 (13.7)	39 (17.2)	28 (12.3)	12 (5.3)	15 (6.6)	
Favorable to AI		4 (28.6)	4 (16.7)	14 (42.4)	14 (63.6)	7 (58.3)	10 (32.3)	18 (46.2)	10 (35.7)	4 (33.3)	7 (46.7)	
Favorable to researchers		10 (71.4)	20 (83.3)	19 (57.6)	8 (36.4)	5 (41.7)	21 (67.7)	21 (53.8)	18 (64.3)	8 (66.7)	8 (53.3)	

Abbreviation: AI, artificial intelligence.

When examining disagreements where the direction of the discrepancy could be adjudicated, the pattern was mixed across items but broadly consistent with a modest AI‐leaning optimism. Disagreements were more frequently favorable to the AI for Item 4 and Item 5, whereas they more often favored researchers for Items 2, 6, 8, and 9. Items 7 and 10 showed a more balanced distribution of discrepancy direction.

Building on these findings, our results can be interpreted alongside the established human interrater reliability of the PEDro scale [5]. Reported item‐level reliability among human raters ranged from fair to substantial (but improved when ratings were generated by consensus panels). For the total score, ICCs were 0.55‐0.56 for individual raters and 0.68 for consensus ratings, supporting the idea that PEDro is most dependable when used in a two‐rater‐plus‐adjudication workflow typical of SRs.

Our findings reinforce that item scoring is not purely mechanical but involves judgment about how methodological features are described and whether they meet the intent of each criterion. Even when researchers follow the same checklist, differences in interpretation, reporting clarity, and contextual assumptions can produce item‐level variability, which is why some degree of residual disagreement is expected in real‐world appraisal workflows. This interpretive component is particularly relevant for items that require weighing adequacy or clarity of reporting rather than simply confirming the presence of a single, unambiguous statement.

However, our disagreement pattern suggests an additional nuance that is distinct from typical human‐human variability. While Maher et al. [5] framed disagreement largely as an issue of occasional missed methodological descriptions or differences in how strictly criteria are interpreted, our results indicate a modest but systematic AI‐leaning optimism, with higher mean total scores and item‐specific directional tendencies. This score overestimation could influence how the credibility of included studies is judged and, consequently, how confidently the overall body of evidence is interpreted. Thus, even when agreement appears high at the item level, a directional bias toward more favorable ratings may have downstream implications that extend beyond isolated scoring discrepancies and warrant caution if AI is used without human oversight.

This implies that AI could be useful as a “third voice” to surface overlooked text or prompt reconsideration of ambiguous criteria, but it should not be treated as an equal‐weight rater in the consensus algorithm at this stage. A pragmatic integration model may therefore be to use AI as an assistive adjudication aid (e.g., flagging candidate justifications, highlighting likely locations of criteria in the text, and prompting targeted human re‐checks on items known to be more interpretive).

Therefore, although AI and researchers show substantial item‐level agreement, the residual discordance is not random and may reflect item‐specific interpretation tendencies that warrant caution when using AI to support PEDro‐based appraisal without human oversight. Future studies should analyze response consistency and explore AI utility in other contexts (e.g., risk of bias assessments or summarizing effect sizes).

## Conflicts of Interest

The authors declare no conflict of interest.

## Supporting information




**Supporting File 1**: jebm70145‐sup‐0001‐SuppMat.docx.
