# Supplementary material for: Analysis of Item‐Level Disagreement Between Researchers and ChatGPT on the PEDro Scale for Assessing the Methodological Quality of Experimental Studies
Source: J Evid Based Med. 2026 Jun 30;19(2):e70145. doi: 10.1111/jebm.70145 (PMC13320110; doi:10.1111/jebm.70145)
Supplement: Supplementary file 1 — Supporting File 1: jebm70145‐sup‐0001‐SuppMat.docx. [file JEBM-19-0-s001.docx]

**Supplementary Material**

**Chat-GPT prompt used**

“I need you to review and fully understand this methodological quality scale (PEDro scale) and apply it to the attached article. Provide the score for each individual item, along with a brief justification for each item.”.

**List of Systematic Reviews**

1. Onan D, Ekizoğlu E, Arıkan H, Taşdelen B, Özge A, Martelletti P. The efficacy of physical therapy and rehabilitation approaches in chronic migraine: a systematic review and meta-analysis. J Integr Neurosci. 2023;22(5):126.

2. Lew J, Kim J, Nair P. Comparison of dry needling and trigger point manual therapy in patients with neck and upper back myofascial pain syndrome: a systematic review and meta-analysis. J Man Manip Ther. 2021;29(3):136-46.

3. Vázquez-Justes D, Yarzábal-Rodríguez R, Doménech-García V, Herrero P, Bellosta-López P. Effectiveness of dry needling for headache: a systematic review. Neurología (Engl Ed). 2022;37(9):806-15.

4. Xie HM, Zhang KX, Wang S, Wang N, Wang N, Li X, et al. Effectiveness of mirror therapy for phantom limb pain: a systematic review and meta-analysis. Arch Phys Med Rehabil. 2022;103(5):988-97.

5. Ferreira RM, Torres RT, Duarte JA, Gonçalves RS. Non-pharmacological and non-surgical interventions for knee osteoarthritis: a systematic review and meta-analysis. Acta Reumatol Port. 2019;44(3):173-217.

6. Melese H, Alamer A, Getie K, Nigussie F, Ayhualem S. Extracorporeal shock wave therapy on pain and foot functions in subjects with chronic plantar fasciitis: systematic review of randomized controlled trials. Disabil Rehabil. 2022;44(18):5007-14.

7. Nahon RL, Silva Lopes JS, Monteiro de Magalhães Neto A. Physical therapy interventions for the treatment of delayed onset muscle soreness (DOMS): systematic review and meta-analysis. Phys Ther Sport. 2021;52:1-12.

8. Lista-Paz A, Bouza Cousillas L, Jácome C, Fregonezi G, Labata-Lezaun N, Llurda-Almuzara L, et al. Effect of respiratory muscle training in asthma: a systematic review and meta-analysis. Ann Phys Rehabil Med. 2023;66(3):101691.

9. Winser S, Chan HK, Chen WK, Hau CY, Leung SH, Leung YH, et al. Effects of therapeutic exercise on disease severity, balance, and functional independence among individuals with cerebellar ataxia: a systematic review with meta-analysis. Physiother Theory Pract. 2023;39(7):1355-75.

10. González-Rubino JB, Vinolo-Gil MJ, Martín-Valero R. Effectiveness of physical therapy in axillary web syndrome after breast cancer: a systematic review and meta-analysis. Support Care Cancer. 2023;31(5):257.
